# Trajectories of symptoms of depression, distress, and resilience in healthcare workers during the COVID-19 pandemic and toward its end in Czechia

**DOI:** 10.1192/j.eurpsy.2024.1752

**Published:** 2024-05-13

**Authors:** Pavla Brennan Kearns, Zsófia Csajbók, Miroslava Janoušková, Matěj Kučera, David Novák, Barbora Fryčová, Marie Kuklová, Jaroslav Pekara, Jana Šeblová, Dominika Seblova

**Affiliations:** 1 Second Faculty of Medicine, Charles University, Prague, Czechia; 2 Faculty of Humanities, Charles University, Prague, Czechia; 3 Third Faculty of Medicine, Charles University, Prague, Czechia; 4 Faculty of Science, Vrije Universiteit Amsterdam, Amsterdam, Netherlands; 5 National Institute of Mental Health, Klecany, Czechia; 6 Department of Demography and Geodemography, Faculty of Science, Charles University, Prague, Czechia; 7 Medical College, Prague, Czechia; 8 Paediatric Emergency Department, Motol University Hospital Prague, Prague, Czechia

**Keywords:** depression, healthcare, pandemic, resilience, stress

## Abstract

**Background and objectives:**

The mental health of healthcare workers (HCWs) may have improved after the COVID-19 pandemic. We aimed to model the trajectories of psychological distress, depressive symptoms, and resilience during the COVID-19 pandemic and toward its end in HCWs in Czechia and investigate, which COVID-19 work stressors were associated with these trajectories.

**Methods:**

The study included 322 HCWs from the Czech arm of the international HEROES Study who participated in an online questionnaire in two waves during the pandemic and one wave toward its end. Growth mixture modeling identified trajectory patterns of depressive symptoms (measured with Patient Health Questionnaire), distress (General Health Questionnaire), and resilience (Brief Resilience Scale). Logistic regression was applied to estimate the association of COVID-19 stressors with mental health trajectories, adjusting for baseline characteristics.

**Results:**

Trajectory classes revealed both high and low depressive symptoms (high in 61% of participants), distress (high in 82% of participants), and resilience (low in 32% of participants). Depressive symptoms and distress trajectories demonstrated the same shape, first increasing during the pandemic and decreasing toward its end, while resilience remained constant. Exposure to COVID-19 stressors, in particular, the experience of stigmatization, discrimination, and violence, was associated with high depressive symptoms and distress trajectories, but not with resilience.

**Conclusions:**

Interventions provided to HCWs during crises such as pandemic should target distress and depressive symptoms and need to address stigmatization, discrimination, and violence.

## Introduction

Healthcare workers (HCWs) faced an elevated risk of emotional strain and mental health issues during the COVID-19 pandemic due to their exposure to the virus and the demands of their profession [1]. In our prior study involving HCWs in Czechia, we observed that the prevalence of depression among HCWs doubled during the pandemic. This increase was primarily attributed to heightened distress, exposure to COVID-19 patient deaths, and direct contact with COVID-19 patients. Conversely, greater resilience and access to adequate personal protective equipment were strongly linked to a reduced occurrence of depression among HCWs [2]. The pandemic may have significantly eroded the resilience of HCWs, due to the inherent unpredictability of infectious diseases, their ability to affect young, previously healthy people, and the instilled fear of contracting the disease among caregivers themselves. Resilience may be viewed as an individual ability to adapt to external stressors like trauma or threats, and resilience in HCWs during pandemics is underpinned by professional identity, collaboration, effective communication, supportive leaders, and potential for growth.

As opposed to a large body of evidence on the trajectories of mental health symptoms in the general population [3–8, 9, 10], it is less understood how the mental health of HCWs changed during the pandemic and toward its end. A study on Italian HCWs found that their mental health improved after the initial peak of the pandemic and the decrease in depressive symptoms was related to being a frontline HCW [11]. However, high depressive symptoms and distress were found to persist among Spanish HCWs over the duration of the pandemic [12]. In HCWs hailing from Northern Ireland, it was predominantly observed that the majority exhibited a trajectory characterized by low depressive symptomatology throughout the pandemic. However, a notable minority, comprising 13–16% of the total, fell into the high-symptom category. Members of this group consistently experienced symptom levels within the moderate-to-severe range, persisting throughout the fluctuating peaks and troughs of the pandemic [13]. In German HCWs during the first year of the pandemic, it was observed that depressive symptoms have risen, but perceived stress did not change over time [14].

Thus far, the findings concerning the trajectories of mental health among HCWs have been inconsistent and have not consistently addressed the evolution of symptoms throughout the entire duration of the pandemic, including the period leading up to its conclusion. Furthermore, although resilience has emerged as a significant predictor of reduced depression and distress symptoms, there remains a gap in understanding whether the resilience levels of individuals have undergone changes over the course of the pandemic. In the present study, we aimed to model the trajectories of psychological distress, depressive symptoms, and resilience during the COVID-19 pandemic and toward its end in HCWs in Czechia and investigate, which COVID-19 work stressors were associated with these trajectories.

## Methods

### Participants

Participants were healthcare and social service workers, including physicians, nurses, paramedics, social workers, and administrative staff in the Czech arm of the international COVID-19 HEalth caRe wOrkErS (HEROES) Study. This global study assessed the pandemic’s impact on their mental health [15]. Data collection used an online questionnaire, starting in Czechia in June 2020 (wave 0: June 24 to August 30;*n* = 1,778) post the first peak. A follow-up was in spring 2021 (wave 1: February 15 to April 31;*n* = 1,840) during the second peak. The last data collection was in fall 2022 toward the end of the pandemic (wave 2: September 15 to November 15, 2022;*n* = 1,451). To be able to model the trajectories of mental health symptoms, at least three measures of the outcome are needed. Therefore, in the present study, we included in total of 322 individuals who participated in all three waves. Not all participants, however, had complete data in all measures (see more information in the Supplementary Material). All participants gave informed consent prior to survey completion. The HEROES Study was approved by the Columbia University Institutional Review Board. The Czech arm of the HEROES Study was approved by the Ethics Committee of the Ministry of Health as well as the Ethical Review Board of the University Hospital Motol, Prague, Czechia. All methods were performed in accordance with relevant guidelines and regulations.

### Depressive symptoms

Depressive symptomatology was quantified employing the Czech version [16] of the Patient Health Questionnaire (PHQ-9), a widely recognized and validated instrument encompassing nine distinct items that gauge the severity of depression [17]. These items encompass inquiries into diminished interest, emotional despondency, sleep disturbances, diminished vitality, alterations in appetite, reduced self-assurance, difficulties in concentration, altered pace, and contemplation of suicidal ideation. Respondents were tasked with indicating the frequency of their experience of these symptoms over the preceding fortnight, with available response options encompassing “not at all” (yielding a score of 0), “several days” (yielding a score of 1), “more than half the days” (yielding a score of 2), and “nearly every day” (yielding a score of 3). Consequently, the cumulative score spanned from 0 to 27, encapsulating the overall extent of depressive symptomatology.

### Distress

Psychological distress was evaluated utilizing the 12-item General Health Questionnaire (GHQ-12) [18], a well-established instrument frequently employed for the assessment of psychological distress within non-clinical populations. Respondents were prompted to gauge the extent, to which they had experienced specific symptoms associated with psychological functioning and mental well-being during the past week, including aspects such as concentration ability, usefulness, feelings of strain, problem-solving capacity, and the capacity to derive satisfaction from day-to-day activities. These responses were subject to a four-point scale, encompassing options denoted as “less than usual,” “no more than usual,” “rather more than usual,” and “much more than usual.” Participants’ score was calculated by reverse coding the negatively phrased items and summing up all items using the Likert scoring method (0–1–2–3), with a potential maximum score of 36 points.

### Resilience

The assessment of resilience was executed through the utilization of the Brief Resilience Scale [19], an instrument designed to capture an individual’s capacity for recuperation in the face of stress. This scale comprises six items, each designed to gauge one’s ability to rebound from challenging circumstances and navigate through stressful events. The items are subject to evaluation using a five-point scale, with response options ranging from “1 = strongly disagree” to “5 = strongly agree.” Notably, the scale encompasses three positively worded items and three negatively worded items. To ensure consistency in scoring, the negatively worded items were reverse-coded. Consequently, the overall resilience score is computed as the mean of these six items, yielding a range of values from one to five points.

### Cumulative exposure to COVID-19 stressors

Cumulative exposure to COVID-19 stressors was assessed throughout all three waves using seven items on individual stressor. The item was counted if the stressor was reported at least once during the follow-up in any wave. They included contact with COVID-19 patients (close contact with suspect or confirmed COVID-19 patient within the last 7 days; yes/no), experience of death due to COVID-19 (close contact at work with someone or caring for a patient who later passed away; yes/no), experience of stigmatization, discrimination, or violence (having felt stigmatized or discriminated against or having experienced violence as a HCW due to the COVID-19 pandemic; yes/no), assignment of new tasks (assignment to a new team or assignment of new functions since the beginning of the pandemic; yes/no), patient prioritization (having had to decide how to prioritize patients with COVID-19; yes/no), insufficient personal protective equipment (yes/no), and low trust in workplace (trusting that the workplace can manage the COVID-19 pandemic; originally options low/moderate/high; here recorded as yes/no). In the end, we created a sum of these seven items (sum of the total exposure to COVID-19 stressors) and divided this variable into low (0–2 points), medium (3–4 points), and high (5–7 points).

### Other characteristics

Participants´ characteristics were chosen as factors associated with the mental health of HCWs during the COVID-19 pandemic and COVID-19 stressors. All characteristics were assessed at baseline, wave 0. In case of missing data, information from a later wave was used. They included age (years), gender (men/women), occupation (physician/nurse or other medical staff/management/other), and chronic physical illness (presence of a chronic physical illness before the pandemic; yes/no).

### Statistical analysis

Trajectories of depressive symptoms, distress, and resilience were created following the recommended guidelines and the most recent advances in growth mixture modeling [20, 21]. Growth mixture modeling (GMM) is a probabilistic technique that extracts distinct longitudinal trajectories of repeated measures variables. The yielded latent class variables will give an approximation of unobserved memberships among the participants following similar patterns. The most widely used GMM model (where the variances of the latent growth factors were held equal across classes) did not yield interpretable class sizes (e.g., subgroup of *n* = 28), did not converge, or obtained negative residual variance for the latent slope factor. Thus, as an alternative method, for the latent trajectories of depressive symptoms, we employed the covariance pattern growth mixture modeling (CPGMM), estimating unique variances and covariances of the latent slopes and intercepts within each extracted class. This method has the advantage of allowing the classes to be unique and it was developed to avoid methodological artifacts [18]. The trajectories of distress and resilience were extracted with Latent Class Growth Models (LCGM; i.e., fixing the variances of the latent slope and intercept factors at zero) as these models yielded more distinctive classes than the CPGMM. The analyses found the 2-class solutions the most parsimonious in all three variables. Extracting more classes (i.e., three classes) was decided against, as the patterns of the three classes were essentially the same (i.e., consistently low, medium, and high levels), with very small class sizes (smallest class was *n* = 58 in depression, *n* = 6 in distress, and *n* = 18 in resilience), and were not supported by model indicators. See further details of the analysis in Supplementary Methods and Supplementary Tables S1–S3.

We compared depressive symptoms, distress, and resilience between waves with repeated measures analysis of variance (ANOVA) with Bonferroni-type post hoc comparisons between the individual waves. We compared the characteristics of participants between classes using independent samples’ *t*-test and chi-squared test. Effect size was expressed by Cohen’s *d* (<0.2 very small; 0.2–0.5 small; 0.5–0.8 moderate; >0.8 large) and Cramer’s *V* (<0.1 very small; 0.1–0.3 small; 0.3–0.5 moderate; >0.5 large). We performed a multivariable analysis, estimating with logistic regression odds ratio (OR) with 95% confidence interval (CI) for the association of exposure to COVID-19 stressors with the trajectory of high depressive symptoms, high distress, and low resilience (separate outcomes in separate models). First, we entered all individual stressors into the model at the same time, adjusting for baseline characteristics (age, gender, occupation, and chronic physical illness). Second, instead of the individual stressors, we entered the variable sum of total exposure to COVID-19 stressors into the model, adjusting for baseline characteristics. Third, instead of the sum of total exposure to COVID-19 stressors, we entered the 3-level variable (low, medium, high) into the model, adjusting for baseline characteristics.

## Results

We studied 322 HCWs (74% women, mean age at baseline 46 years), from whom 36% were physicians, 36% nurses or other medical staff, and 17% in management (Table 1). Over the three waves of the follow-up, 60% of them have had contact with COVID-19 patients, 48% had the experience of death due to COVID-19, 41% experienced stigmatization, discrimination, or violence, 48% underwent an assignment of new tasks, 20% had to prioritize patients, 42% reported insufficient personal protective equipment, and 28% low trust in their workplace.

**Table 1.** Characteristics of participants (*n* = 322)

Baseline characteristics	Distribution
Age, years, mean ± SD	45.5 ± 11.4
Women, *n* (%)	239 (74.2)
Occupation, *n* (%)	
Physician	116 (36.0)
Nurse or other medical staff	115 (35.7)
Management	54 (16.8)
Other	37 (11.5)
Physical illness, *n* (%)	87 (27.5)
Cumulative exposure to COVID–19 stressors, *n* (%)	
Contact with COVID–19 patients	191 (59.5)
Experience of death due to COVID–19	155 (48.3)
Experience of stigmatization, discrimination, or violence	133 (41.3)
Assignment of new tasks	154 (47.8)
Patient prioritization	65 (20.4)
Insufficient personal protective equipment	132 (41.6)
Low trust in workplace	91 (28.3)
Total exposure to COVID–19 stressors, mean ± SD	2.9 ± 1.7
Low, *n* (%)	146 (45.3)
Medium, *n* (%)	117 (36.3)
High, *n* (%)	59 (18.3)

Abbreviation: SD, standard deviation.

Depressive symptoms differed between waves (*F*[2, 478] = 14.0, *p* < .001, *η_p_*^2^ = .1). In wave 0, depressive symptoms were distributed around the mean of 4.2 (± standard deviation 4.0), then increased in wave 1 (5.5 ± 4.7, *p* < .001) and again decreased in wave 2 (4.5 ± 4.3, *p* = .001). A similar pattern was observed for distress (*F*[2, 498] = 40.7, *p* < .001, *η_p_*^2^ = .1). In wave 0, the average distress score reached 11.6 ± 4.6, then increased in wave 1 (13.9 ± 5.5, *p* < .001) and again decreased in wave 2 (11.2 ± 4.5, *p* < .001). On the contrary, resilience remained constant across waves (mean 3.4 ± 0.7 in waves 0–2; *F*[2, 478] = .6, *p* = .4, *η_p_*^2^ < .1).

**Figure 1.**
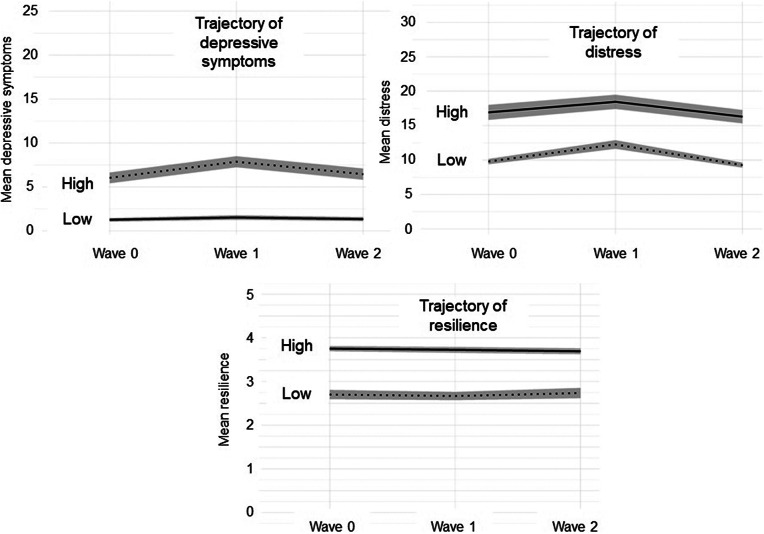
Classes of trajectories of mental health symptoms.

There were two classes of the development of depressive symptoms, distress and resilience over time, for each outcome, there was a class of high symptoms and low symptoms (Figure 1). Low depressive symptoms trajectory was present in 39% of the sample, while 61% had high depressive symptoms trajectory. However, it needs to be acknowledged that when considering the cut-off criteria based on PHQ, the high depressive symptoms trajectory corresponds largely to mild or moderate symptomatology, while the low symptoms trajectory group includes largely asymptomatic individuals. However, to ensure readability, here we used the terms high symptoms and low symptoms. Both classes had the same trajectory shape – first, the symptoms increased between wave 0 and wave 1 and then decreased between wave 1 and wave 2. Low distress trajectory was present in 72% of the sample, while 28% had high distress trajectory. Similar to the classes of depressive symptoms was the trajectory of distress – first, there was an increase in distress from wave 0 and wave 1, followed by a decrease between wave 1 and wave 2. Concerning resilience, 68% of the sample was classified with high resilience trajectory and 32% with low resilience trajectory. Within both classes, there were no changes in resilience over time.

There were small differences in participants´ characteristics between the trajectory classes (Table 2). In particular, younger people, women, people with the experience of stigmatization, discrimination, or violence, and those with greater exposure to COVID-19 stressors had more often high depressive symptoms. People with the experience of stigmatization, discrimination or violence, insufficient protective equipment, and greater exposure to COVID-19 stressors had more often high distress. Women, people with the experience of stigmatization, discrimination, or violence and low trust in their workplace had more often low resilience. Furthermore, those with low resilience were also more depressed and distressed than those with high resilience.

**Table 2.** Differences in participants’ characteristics between classes

	Depressive symptoms			Distress			Resilience		
Baseline characteristics	Low (*n* = 124)	High (*n* = 196)	d/V	Low (*n* = 231)	High (*n* = 90)	d/V	Low (*n* = 102)	High (*n* = 217)	d/V
Age, years, mean ± SD	**47.8 ± 11.1**	**44.1 ± 11.3**	**0.33**	45.9 ± 11.5	44.6 ± 11.2	0.12	44.2 ± 10.9	46.2 ± 11.5	0.18
Women, *n* (%)	**80 (64.5)**	**157 (80.1)**	**0.17**	165 (71.4)	73 (81.1)	0.10	**84 (82.4)**	**153 (70.5)**	**0.13**
Occupation, *n* (%)									
Physician	45 (36.3)	70 (35.7)	0.03	85 (36.8)	30 (33.3)	0.08	33 (32.4)	82 (37.8)	0.06
Nurse or other medical	45 (36.3)	69 (35.2)		81 (35.1)	34 (37.8)		40 (39.2)	73 (33.6)	
Management	21 (16.9)	33 (16.8)		36 (15.6)	18 (20.0)		18 (17.6)	36 (16.6)	
Other	13 (10.5)	24 (12.2)		29 (12.6)	8 (8.9)		11 (10.8)	26 (12.0)	
Physical illness, *n* (%)	32 (26.0)	55 (28.5)	0.03	60 (26.4)	27 (30.3)	0.04	31 (31.0)	56 (25.9)	0.05
Cumulative exposure to COVID–19 stressors, *n* (%)									
Contact with COVID–19 patients	72 (58.1)	118 (60.5)	0.02	130 (56.3)	60 (67.4)	0.10	56 (55.4)	133 (61.3)	0.06
Experience of death due to COVID–19	53 (43.1)	102 (52.0)	0.09	104 (45.2)	51 (56.7)	0.10	44 (43.1)	110 (50.9)	0.07
Experience of stigmatization, discrimination, or violence	**40 (32.3)**	**93 (47.4)**	**0.15**	**85 (36.8)**	**48 (53.3)**	**0.15**	**51 (50.0)**	**82 (37.8)**	**0.12**
Assignment of new tasks	51 (41.1)	102 (52.0)	0.11	109 (47.2)	44 (48.9)	0.02	50 (49.0)	102 (47.0)	0.02
Patient prioritization	21 (17.2)	44 (22.6)	0.06	46 (20.2)	19 (21.1)	0.10	21 (20.6)	43 (20.1)	0.01
Insufficient personal protective equipment	44 (36.1)	88 (45.6)	0.09	**86 (37.7)**	**46 (52.3)**	**0.13**	**53 (53.0)**	**78 (36.4)**	**0.16**
Low trust in workplace	34 (27.4)	57 (29.1)	0.02	59 (25.5)	32 (35.6)	0.10	**38 (37.3)**	**53 (24.4)**	**0.13**
Total exposure to COVID–19 stressors, mean ± SD	**2.5 ± 1.6**	**3.1 ± 1.7**	**0.33**	**2.7 ± 1.7**	**3.3 ± 1.7**	**0.40**	3.1 ± 1.7	2.8 ± 1.6	0.18
Low, *n* (%)	65 (52.4)	79 (40.3)	0.12	**113 (48.9)**	**32 (35.6)**	**0.14**	44 (43.1)	100 (46.1)	0.04
Medium, *n* (%)	39 (31.5)	78 (39.8)		**82 (35.5)**	**35 (38.9)**		37 (36.3)	80 (36.9)	
High, *n* (%)	20 (16.1)	39 (19.9)		**36 (15.6)**	**23 (25.6)**		21 (20.6)	37 (17.1)	
Depressive symptoms, mean ± SD									
Wave 0	**1.3 ± 1.2**	**6.0 ± 4.2**	**1.43**	**2.7 ± 2.5**	**8.0 ± 4.8**	**1.62**	**6.3 ± 5.0**	**3.2 ± 3.1**	**0.82**
Wave 1	**1.5 ± 1.4**	**7.9 ± 4.3**	**1.83**	**4.1 ± 3.9**	**8.9 ± 4.6**	**1.15**	**7.8 ± 4.9**	**4.3 ± 4.0**	**0.83**
Wave 2	**1.4 ± 1.3**	**6.5 ± 4.3**	**1.50**	**2.8 ± 2.7**	**8.5 ± 4.6**	**1.68**	**6.5 ± 5.0**	**3.5 ± 3.4**	**0.77**
Distress, mean ± SD									
Wave 0	**9.1 ± 2.9**	**13.5 ± 5.0**	**1.04**	**9.8 ± 2.9**	**16.9 ± 5.0**	**1.98**	**14.1 ± 5.5**	**10.7 ± 4.1**	**0.75**
Wave 1	**9.9 ± 3.4**	**16.6 ± 4.8**	**1.54**	**12.3 ± 4.6**	**18.5 ± 4.7**	**1.33**	**16.8 ± 5.3**	**12.6 ± 4.9**	**0.85**
Wave 2	**8.4 ± 2.2**	**13.2 ± 4.7**	**1.22**	**9.3 ± 2.7**	**16.3 ± 4.5**	**2.15**	**13.6 ± 5.2**	**10.2 ± 3.8**	**0.79**
Resilience, mean ± SD									
Wave 0	**3.7 ± 0.6**	**3.2 ± 0.7**	**0.71**	**3.6 ± 0.6**	**3.0 ± 0.7**	**0.82**	**2.7 ± 0.5**	**3.8 ± 0.4**	**2.29**
Wave 1	**3.7 ± 0.6**	**3.2 ± 0.6**	**0.90**	**3.5 ± 0.6**	**3.0 ± 0.7**	**0.73**	**2.7 ± 0.5**	**3.7 ± 0.5**	**2.27**
Wave 2	**3.7 ± 0.6**	**3.2 ± 0.7**	**0.82**	**3.6 ± 0.6**	**3.0 ± 0.7**	**0.96**	**2.7 ± 0.6**	**3.7 ± 0.5**	**1.88**

*Note:* Differences between groups were assessed using independent samples *t*-test or chi-squared test. Significant results (*p* < 0.05) are in bold.

Table 3 presents the association of cumulative exposure to COVID-19 stressors with the trajectories classes. Looking at the stressors separately, only the experience of stigmatization, discrimination, or violence was associated with the high depressive symptoms trajectory (OR 1.92; 95% CI 1.15; 3.24) and high distress trajectory (OR 1.73; 95% CI 1.01; 2.97). Insufficient personal protective equipment was associated with the low resilience trajectory (OR 1.99; 95% CI 1.16; 3.42). Considering the total cumulative exposure to COVID-19 stressors, the sum of the stressors was associated with greater odds of belonging to the high depressive symptoms trajectory (OR 1.29; 95% CI 1.10; 1.52). When compared to the low total exposure, only the medium total exposure was related to greater odds of belonging to the high depressive symptoms trajectory (OR 1.88; 95% 1.10; 3.28), while the odds for the high exposure were lower and did not reach statistical significance. The sum of the stressors was also associated with greater odds of high distress trajectory (OR 1.32; 95% CI 1.12; 1.56), showing a dose–response pattern as the high total exposure was related to the greatest odds of belonging to the high distress trajectory class (OR 2.53; 95% CI 1.24; 5.18). Total exposure to COVID-19 stressors was not related to trajectories of resilience.

**Table 3.** Association of cumulative exposure to COVID-19 stressors with trajectories of mental health

	OR (95% CI)		
	High depressive symptoms trajectory	High distress symptoms trajectory	Low resilience trajectory
A) Individual stressors			
Contact with COVID–19 patients	1.00 (0.57; 1.76)	1.57 (0.85; 2.94)	0.89 (0.50; 1.59)
Experience of death due to COVID–19	1.47 (0.85; 2.55)	1.46 (0.81; 2.64)	0.68 (0.38; 1.20)
Experience of stigmatization, discrimination, or violence	**1.92 (1.15; 3.24)**	**1.73 (1.01; 2.97)**	1.37 (0.81; 2.32)
Assignment of new tasks	1.32 (0.79; 2.22)	0.78 (0.44; 1.36)	1.07 (0.63; 1.83)
Patient prioritization	1.14 (0.57; 2.31)	0.97 (0.47; 1.95)	1.12 (0.55; 2.25)
Insufficient personal protective equipment	1.42 (0.84; 2.43)	1.72 (0.99; 3.02)	**1.99 (1.16; 3.42)**
Low trust in workplace	0.96 (0.54; 1.70)	1.37 (0.76; 2.44)	1.37 (0.78; 2.39)
B) Total exposure to stressors			
Sum of stressors	**1.29 (1.10; 1.52)**	**1.32 (1.12; 1.56)**	1.15 (0.98; 1.34)
Exposure to stressors			
Low	Reference		
Medium	**1.88 (1.10; 3.28)**	1.58 (0.89; 2.85)	1.13 (0.65; 1.97)
High	1.60 (0.81; 3.24)	**2.53 (1.24; 5.18)**	1.34 (0.67; 2.68)

*Note:* In part A), all variables on individual stressors were entered into the model at the same time and the model was additionally adjusted for age, gender, occupation, and chronic physical illness. In part B), the variable sum of stressors (continuous) was entered alone into the model, which was adjusted for age, gender, occupation, and chronic physical illness. The variable exposure to stressors (categorical) was entered alone into the model, which was adjusted for age, gender, occupation, and chronic physical illness. CI, confidence interval; OR, odds ratio.

Results in bold are statistically significant.

## Discussion

Our analysis unveiled that, during the initial wave of the pandemic, a substantial 61% of HCWs exhibited pronounced depressive symptoms, a figure that escalated as the pandemic progressed, subsequently receding upon its abatement. The remaining 39% of HCWs manifested comparatively lower levels of depressive symptoms; nevertheless, they too witnessed an increase and subsequent decrease in symptomatology. Intriguingly, only experiences of stigmatization, discrimination, or violence were found to independently correlate with the trajectory characterized by heightened depressive symptoms. While overall exposure to COVID-19-related stressors demonstrated an association with the trajectory of elevated depressive symptoms, this relationship did not adhere to a linear dose–response pattern. Moreover, 28% of individuals experienced high distress, whereas the majority, constituting 72%, reported a trajectory marked by low distress levels. The trajectories of distress mirrored those observed for depressive symptoms. Similarly, only reported encounters with stigmatization, discrimination, or violence bore independent association with the trajectory marked by elevated distress. The cumulative exposure to COVID-19-related stressors exhibited a distinct pattern, displaying a dose–response relationship with the trajectory characterized by heightened distress symptoms. Lastly, our findings indicated that 32% of the study participants exhibited low levels of resilience, with the majority, accounting for 68%, demonstrating high resilience. Remarkably, resilience levels remained relatively stable throughout the follow-up period. We observed that low resilience was linked to reports of inadequate personal protective equipment, although it did not correlate with the overall extent of exposure to COVID-19-related stressors.

A large body of evidence has pointed to the poor mental health of HCWs during the pandemic [22–25]. Here we uniquely show great improvements in the symptoms of distress and depression in HCWs toward the end of the pandemic. We found that the trajectory characterized by high levels of depressive symptoms was strongly linked to experiences of stigmatization, discrimination, or violence. This observation is consistent with existing knowledge indicating that experienced stigma [26], discrimination [27], and workplace violence [28, 29] are established risk factors for depression. Moreover, this connection extends to the long-term association of discrimination with subsequent depression [30]. While the pathway from exposure to discrimination due to COVID-19 to depression has not been explicitly described, a social cognitive model developed to understand racial discrimination [31] could be applied for explanatory purposes. This model, inclusive of relational schemas reflecting concerns about rejection and invalidation, social vigilance, and mistrust, serves as a mediator in the link between discrimination and depression [31]. In the context of the COVID-19 pandemic, HCWs who have experienced discrimination may harbor feelings of rejection, vigilance, and mistrust within society, thereby contributing to the development of depressive symptoms. Notably, although the trajectory characterized by high levels of depressive symptoms demonstrated an association with overall exposure to COVID-19-related stressors, this relationship did not exhibit a linear dose–response pattern, suggesting a likely absence of causality. This phenomenon can be elucidated by the pandemic’s capacity to induce an increased prevalence of depression across the Czech general population [32], precipitating significant mental health consequences irrespective of the level of exposure to COVID-19 stressors. This prompts questions regarding the threshold at which stressors become clinically significant. It is, therefore, likely that there are confounding factors at play, which we did not take into account.

The trajectory of psychological distress exhibited an upward trend during the initial wave, which coincided with the lockdown measures implemented in Czechia at the peak of the pandemic. This trend aligns with the findings of a study conducted in Australia [33]. Similarly as to depressive symptoms, the trajectory characterized by high distress levels was also associated with experiences of stigmatization, discrimination, or violence. These distressing encounters represent significant risk factors for psychological distress and may exert enduring effects on psychological well-being that extend beyond the pandemic’s immediate impact. HCWs, having experienced such stressors, may retain concerns that others will treat them similarly to their experiences during the outbreak. Moreover, the trajectory marked by high distress levels exhibited a dose–response relationship with the overall exposure to COVID-19 stressors, implying a potential causal link and suggesting that the stressors cumulatively meant a more substantial effect than each individually. This observation aligns with findings from a systematic review [34] indicating that risk factors for psychological distress during infectious disease outbreaks primarily involve infection exposure factors, such as contact with infected individuals or colleagues. Frontline HCWs [35] emerge as particularly vulnerable to distress.

Interestingly, resilience levels remained relatively stable throughout the pandemic. Notably, the low resilience trajectory did not exhibit an association with the overall exposure to COVID-19 stressors. This supports the idea that the measure of resilience we used is trait-like and does not capture a dynamic state [36]. Our results therefore cannot suggest that HCWs should be offered interventions that would increase their resilience [37]. In another study, resilience scores did not change significantly during the COVID-19 pandemic either [38]. To et al. found significant associations of resilience with physical activity and psychological distress, suggesting that future interventions to enhance or nurture resilience should be particularly targeted at people identified as at risk of psychological distress [38]. In our study, we found that the low resilience trajectory was associated with reporting insufficient personal protective equipment. This finding is corroborated by an Italian study [39], which observed that HCWs satisfied with their personal protective equipment had higher levels of resilience. HCWs possess an understanding of the protective properties of different personal protective equipment and maintain confidence that inadequate personal protective equipment offers no protection against the risk of infection [40].

Several strengths and limitations need to be mentioned. The observation of changes in mental health over three waves of COVID-19 pandemic in Czechia (longitudinal design) belongs to the strengths of the study. The comparison among trajectory groups provides valuable insight into the relationship between the level of exposure, both cumulative and individually, and the severity of mental health deterioration. On the other hand, self-reporting used in the data collection may introduce information bias. Also, the relatively small sample size (*n* = 322) is a limitation of this study. This study is also limited by a large dropout of the sample during the assessments as individuals who are likely healthier and more motivated may have remained in the study. Furthermore, this study is based only on one nation and its results can be influenced by specific Czech population mental health and conditions and cannot be generalized. In addition, the scale used in our study may not be optimal for measuring resilience. Although several resilience scales have been published [41], each tends to include different traits and in general these scales fail to explain why so many of the empirically identified correlates of resilient outcomes are not included in the personality, or why these factors may nonetheless still influence resilient outcomes. Most critically, although resilience scales are generally correlated with health and well-being, they do not hold up to their promise when tested in longitudinal or prospective research. In the end, this study did not consider a specific occupational field of HCWs, which could have provided more nuanced results.

To conclude, policymakers should address stigmatization, discrimination, and violence in healthcare and make safe and supportive work environments to protect HCWs. Destigmatization could be realized through communication, open dialogue, and promotion of reliable sources of information. Self-help training and psychological support should be available in healthcare facilities.

## Supporting information

Brennan Kearns et al. supplementary material Brennan Kearns et al. supplementary material

## Supplementary material

The supplementary material for this article can be found at http://doi.org/10.1192/j.eurpsy.2024.1752.

## References

[1] Rangappa SB, Avula S. Mental health challenges in health care workers during COVID pandemic. Eur Psychiatry. 2023;66(S1):S602.

[2] Cermakova P, Fryčová B, Novák D, Kuklová M, Wolfová K, Kučera M, et al. Depression in healthcare workers during COVID-19 pandemic: results from Czech arm of HEROES study. Sci Rep. 2023;13(1):12430. 37528158 10.1038/s41598-023-39735-w PMC10394070

[3] Batterham PJ, Calear AL, McCallum SM, Morse AR, Banfield M, Farrer LM, et al. Trajectories of depression and anxiety symptoms during the COVID‐19 pandemic in a representative Australian adult cohort. Med J Aust. 2021;214(10):462–8. 33899939 10.5694/mja2.51043 PMC8207103

[4] Mooldijk SS, Dommershuijsen LJ, de Feijter M, Luik AI. Trajectories of depression and anxiety during the COVID-19 pandemic in a population-based sample of middle-aged and older adults. J Psychiatr Res. 2022;149:274–80. 35305381 10.1016/j.jpsychires.2022.03.002 PMC8906533

[5] Hawes MT, Szenczy AK, Olino TM, Nelson BD, Klein DN. Trajectories of depression, anxiety and pandemic experiences; a longitudinal study of youth in New York during the spring-summer of 2020. Psychiatry Res. 2021;298:113778. 33550176 10.1016/j.psychres.2021.113778 PMC9754702

[6] van Loon AW, Creemers HE, Vogelaar S, Saab N, Miers AC, Westenberg PM, et al. Trajectories of adolescent perceived stress and symptoms of depression and anxiety during the COVID-19 pandemic. Sci Rep. 2022;12(1):15957. 36153394 10.1038/s41598-022-20344-y PMC9509354

[7] Piumatti G, Levati S, Amati R, Crivelli L, Albanese E, Group CITW. Trajectories of depression, anxiety and stress among adults during the COVID-19 pandemic in southern Switzerland: the Corona Immunitas Ticino cohort study. Public Health. 2022;206:63–9. 35381519 10.1016/j.puhe.2022.02.005 PMC8825315

[8] Riehm KE, Holingue C, Smail EJ, Kapteyn A, Bennett D, Thrul J, et al. Trajectories of mental distress among US adults during the COVID-19 pandemic. Ann Behav Med. 2021;55(2):93–102. 33555336 10.1093/abm/kaaa126 PMC7929474

[9] Fancourt D, Steptoe A, Bu F. Trajectories of anxiety and depressive symptoms during enforced isolation due to COVID-19 in England: a longitudinal observational study. Lancet Psychiatry. 2021;8(2):141–9. 33308420 10.1016/S2215-0366(20)30482-X PMC7820109

[10] Joshi D, Gonzalez A, Griffith L, Duncan L, MacMillan H, Kimber M, et al. The trajectories of depressive symptoms among working adults during the COVID-19 pandemic: a longitudinal analysis of the InHamilton COVID-19 study. BMC Public Health. 2021;21:1–10. 34666722 10.1186/s12889-021-11900-8 PMC8526051

[11] Rossi R, Socci V, Jannini TB, Pacitti F, Siracusano A, Rossi A, et al. Mental health outcomes among Italian health care workers during the COVID-19 pandemic. JAMA Netw Open. 2021;4(11):e2136143. 34817580 10.1001/jamanetworkopen.2021.36143 PMC8613589

[12] Mediavilla R, Fernández-Jiménez E, Martinez-Morata I, Jaramillo F, Andreo-Jover J, Morán-Sánchez I, et al. Sustained negative mental health outcomes among healthcare workers over the first year of the COVID-19 pandemic: a prospective cohort study. Int J Public Health. 2022;67:1604553. 35814735 10.3389/ijph.2022.1604553 PMC9266625

[13] Jordan J-A, Shannon C, Browne D, Carroll E, Maguire J, Kerrigan K, et al. Healthcare staff mental health trajectories during the COVID-19 pandemic: findings from the COVID-19 staff wellbeing survey. BJPsych Open. 2023;9(4):e112. 37345555 10.1192/bjo.2023.497 PMC10305016

[14] Hoffmann S, Schulze S, Löffler A, Becker J, Hufert F, Gremmels H-D, et al. Did the prevalence of depressive symptoms change during the COVID-19 pandemic? A multilevel analysis on longitudinal data from healthcare workers. Int J Soc Psychiatry. 2024;70:87–98. 37671660 10.1177/00207640231196737 PMC10860357

[15] Mascayano F, Van der Ven E, Moro MF, Schilling S, Alarcón S, Al Barathie J, et al. The impact of the COVID-19 pandemic on the mental health of healthcare workers: study protocol for the COVID-19 HEalth caRe wOrkErS (HEROES) study. Soc Psychiatry Psychiatr Epidemiol. 2022;57(3):633–45. 35064280 10.1007/s00127-021-02211-9 PMC8782684

[16] Daňsová P, Masopustová Z, Hanáčková V, Kicková K, Korábová I. Metoda patient health questionnaire-9: Česká Verze. Ceskoslovenska Psychologie. 2016;60(5):468.

[17] Löwe B, Kroenke K, Herzog W, Gräfe K. Measuring depression outcome with a brief self-report instrument: sensitivity to change of the patient health questionnaire (PHQ-9). J Affect Disord. 2004;81(1):61–6. 15183601 10.1016/S0165-0327(03)00198-8

[18] Goldberg DP, Gater R, Sartorius N, Ustun TB, Piccinelli M, Gureje O, et al. The validity of two versions of the GHQ in the WHO study of mental illness in general health care. Psychol Med. 1997;27(1):191–7. 9122299 10.1017/s0033291796004242

[19] Smith BW, Dalen J, Wiggins K, Tooley E, Christopher P, Bernard J. The brief resilience scale: assessing the ability to bounce back. Int J Behav Med. 2008;15:194–200. 18696313 10.1080/10705500802222972

[20] McNeish D, Harring J. Covariance pattern mixture models: eliminating random effects to improve convergence and performance. Behav Res Methods. 2020;52:947–79. 31512175 10.3758/s13428-019-01292-4

[21] Van De Schoot R, Sijbrandij M, Winter SD, Depaoli S, Vermunt JK. The GRoLTS-checklist: guidelines for reporting on latent trajectory studies. Struct Equ Model Multidiscip J. 2017;24(3):451–67.

[22] Vizheh M, Qorbani M, Arzaghi SM, Muhidin S, Javanmard Z, Esmaeili M. The mental health of healthcare workers in the COVID-19 pandemic: a systematic review. J Diabetes Metab Disord. 2020;19(2):1967–78. doi:10.1007/s40200-020-00643-9. 33134211 PMC7586202

[23] Saragih ID, Tonapa SI, Saragih IS, Advani S, Batubara SO, Suarilah I, et al. Global prevalence of mental health problems among healthcare workers during the Covid-19 pandemic: a systematic review and meta-analysis. Int J Nurs Stud. 2021;121:104002. 34271460 10.1016/j.ijnurstu.2021.104002 PMC9701545

[24] Ghahramani S, Kasraei H, Hayati R, Tabrizi R, Marzaleh MA. Health care workers’ mental health in the face of COVID-19: a systematic review and meta-analysis. Int J Psychiatry Clin Pract. 2023;27(2):208–17. doi:10.1080/13651501.2022.2101927. 35875844

[25] Umbetkulova S, Kanderzhanova A, Foster F, Stolyarova V, Cobb-Zygadlo D. Mental health changes in healthcare workers during COVID-19 pandemic: a systematic review of longitudinal studies. Eval Health Prof. 2023;47:11. doi:10.1177/01632787231165076. 37143216 PMC10160822

[26] Uphoff EP, Lombardo C, Johnston G, Weeks L, Rodgers M, Dawson S, et al. Mental health among healthcare workers and other vulnerable groups during the COVID-19 pandemic and other coronavirus outbreaks: a rapid systematic review. PLoS One. 2021;16(8):e0254821. 34347812 10.1371/journal.pone.0254821 PMC8336853

[27] Lee S, Waters SF. Asians and Asian Americans’ experiences of racial discrimination during the COVID-19 pandemic: impacts on health outcomes and the buffering role of social support. Stigma Health. 2021;6(1):70.

[28] Hansen ÅM, Hogh A, Persson R. Frequency of bullying at work, physiological response, and mental health. J Psychosom Res. 2011;70(1):19–27. 21193097 10.1016/j.jpsychores.2010.05.010

[29] Chowdhury SR, Kabir H, Mazumder S, Akter N, Chowdhury MR, Hossain A. Workplace violence, bullying, burnout, job satisfaction and their correlation with depression among Bangladeshi nurses: a cross-sectional survey during the COVID-19 pandemic. PLoS One. 2022;17(9):e0274965. 36137141 10.1371/journal.pone.0274965 PMC9499253

[30] Narita Z, Okubo R, Sasaki Y, Takeda K, Ohmagari N, Yamaguchi K, et al. Association of COVID-19-related discrimination with subsequent depression and suicidal ideation in healthcare workers. J Psychiatr Res. 2023;159:153–8. doi:10.1016/j.jpsychires.2023.01.025. 36731380 PMC9849914

[31] Mikrut EE, Keating LH, Barnwell PV, Cioffi L, Vega D, Contrada RJ, et al. Pathways from exposure to racial/ethnic discrimination to depression: testing a social-cognitive model. Soc Sci Med. 1982;292:114558. doi:10.1016/j.socscimed.2021.114558. 34891028

[32] Winkler P, Mohrova Z, Mlada K, Kuklova M, Kagstrom A, Mohr P, et al. Prevalence of current mental disorders before and during the second wave of COVID-19 pandemic: an analysis of repeated nationwide cross-sectional surveys. J Psychiatr Res. 2021;139:167–71. doi:10.1016/j.jpsychires.2021.05.032. 34062293 PMC8769682

[33] Botha F, Morris RW, Butterworth P, Glozier N. Trajectories of psychological distress over multiple COVID-19 lockdowns in Australia. SSM Popul Health. 2023;21:101315. doi:10.1016/j.ssmph.2022.101315. 36530365 PMC9742066

[34] Sirois FM, Owens J. Factors associated with psychological distress in health-care workers during an infectious disease outbreak: a rapid systematic review of the evidence. Front Psych. 2020;11:589545. doi:10.3389/fpsyt.2020.589545. PMC7876062 33584364

[35] De Kock JH, Latham HA, Leslie SJ, Grindle M, Munoz S-A, Ellis L, et al. A rapid review of the impact of COVID-19 on the mental health of healthcare workers: implications for supporting psychological well-being. BMC Public Health. 2021;21(1):1–18. 33422039 10.1186/s12889-020-10070-3 PMC7794640

[36] Kuldas S, Foody M. Neither resiliency-trait nor resilience-state: transactional resiliency/e. Youth Soc. 2022;54(8):1352–76.

[37] Abegglen S, Greif R, Fuchs A, Berger-Estilita J. COVID-19–related trajectories of psychological health of acute care healthcare professionals: A 12-month longitudinal observational study. Front Psychol. 2022;13:900303. 35846720 10.3389/fpsyg.2022.900303 PMC9280365

[38] To QG, Vandelanotte C, Cope K, Khalesi S, Williams SL, Alley SJ, et al. The association of resilience with depression, anxiety, stress and physical activity during the COVID-19 pandemic. BMC Public Health. 2022;22(1):491. 35279118 10.1186/s12889-022-12911-9 PMC8917786

[39] Trotzky D, Aizik U, Mosery J, Carady N, Tavori G, Cohen A, et al. Resilience of hospital staff facing COVID-19 pandemic: lessons from Israel. Front Public Health. 2023;11:1050261. 37064690 10.3389/fpubh.2023.1050261 PMC10102590

[40] Lisi L, Ciaffi J, Bruni A, Mancarella L, Brusi V, Gramegna P, et al. Levels and factors associated with resilience in Italian healthcare professionals during the COVID-19 pandemic: a web-based survey. Behav Sci (Basel). 2020;10(12):183. doi:10.3390/bs10120183. 33260390 PMC7760580

[41] Bonanno GA, Chen S, Bagrodia R, Galatzer-Levy IR. Resilience and disaster: flexible adaptation in the face of uncertain threat. Ann Rev Psychol. 2023;75:573. 37566760 10.1146/annurev-psych-011123-024224

