# Supplementary material for: Trajectories of symptoms of depression, distress, and resilience in healthcare workers during the COVID-19 pandemic and toward its end in Czechia
Source: Eur Psychiatry. 2024 May 13;67(1):e49. doi: 10.1192/j.eurpsy.2024.1752 (PMC11441339; doi:10.1192/j.eurpsy.2024.1752)
Supplement: Brennan Kearns et al. supplementary material [file S0924933824017528sup001.docx]

**SUPPLEMENT**

*Growth Mixture Modelling*

First, we fitted latent growth models on the depressive symptoms, distress, and resilience variables across the three waves of measurement. We estimated missing values with the full information maximum likelihood method (FIML). We included participants who took part in all three waves, but we did not restrict the sample to participants who had complete responses in all three waves. We did so because the FIML method used for missing data handling has very good performance in Structural Equation Modeling methods, even in small sample sizes [1]. FIML is also the most widely applied method in Growth Mixture Modeling studies [2]. Re-running the model with full data coverage yields essentially the same results as patterns obtained with the FIML method [3, 4], therefore we argue that the trade-off between restricting the sample to full data coverage versus using all available information is best dealt with if we do not further decrease the sample size unnecessarily.

To help the models converge, we divided the depressive symptoms and distress scores by 10 thereby keeping the variances between 1 to 10 (Table S1), following the common practice when using the Mplus software [5]. The latent growth model did not yield acceptable model fit on the depressive symptoms and distress variables, but yielded excellent model fit on resilience (X^2^(1) = 1.401, RMSEA = 0.035 (90% CI 0.000, 0.158), CFI = 0.999, TLI = 0.997, SRMR = 0.012). An alternative model, the latent base growth model was fitted on the depressive symptoms and distress variables. This method allowed for freely estimated trajectory patterns by fixing the two ends of the trajectory slopes at zero and one, and freely estimating the slope in the middle instead of fixing them at zero, one, and two as in a linear growth model. Here, the loading on the latent slope factor of wave 0 was fixed at 0, while the loading of wave 2 was fixed at 1, and the loading of wave 1 was estimated freely. Being a just identified model, the model yielded excellent model fit on depressive symptoms, but the model did not converge on distress. To inspect if this affects the mixture modelling results, we submitted the latent base growth model into the mixture modelling for both depressive symptoms and distress.

The most widely used Growth Mixture Model (GMM, where the variances of the latent growth factors were held equal across classes) did not yield interpretable class sizes (e.g., subgroup of n = 28), did not converge, or obtained negative residual variance for the latent slope factor. Therefore, subsequently, we extracted latent classes with the covariance pattern growth mixture modelling method (CPGMM [6]) that freely estimates unique variances of the latent growth factors in each class. This method has the advantage of allowing the classes to be unique and it was developed to avoid methodological artefacts [6]. The CPGMM model suggested two distinct classes for depressive symptoms, distress, and resilience. However, the model yielded relatively low distinctiveness in distress (mean difference between the intercepts of the two classes was only 0.500) and in resilience (mean difference between the intercepts of the two classes was only 0.516). For this reason, latent class growth models were estimated for both distress and resilience (with variances fixed at zero in each class).

To test if extracting k classes was more parsimonious than extracting k-1 classes, we obtained the Akaike Information Criterion (AIC [7]), Bayesian Information Criterion (BIC [8]), the Vuong-Lo-Mendell-Rubin Likelihood Ratio Test (VLMR LRT [9]), the Lo-Mendell-Rubin adjusted likelihood ratio test (LMR ALRT [10]), the parametric bootstrapped likelihood ratio test (BLRT [11]), and entropy. In the 2-class solutions, in all three measures (i.e., depressive symptoms, distress, and resilience) all the VLMR LRT, LMR ALRT, and BLRT tests were significant (p < 0.05), indicating a better model in the 2-class models versus in the 1-class models (with a significant test indicating better k-class model in comparison to the k-1 class solution).

The extraction of three classes in depressive symptoms was supported by significant VLMR LRT and LMR ALRT tests, however, the BLRT test did not replicate the best loglikelihood value for the 3-class model (meaning p < 0.001 may not be trustworthy) even after increasing the LRT random starts to 0 0 1000 200. The 3-class model’s AIC (354.227) and BIC (455.972) were smaller than the 2-class solutions, however, the result’s interpretability did not support the use of the 3-class model. That is, the patterns of the three classes were essentially the same (i.e., consistently low, medium, and high levels; or in other words, the additional classes only yielded differing intercepts but no significant slopes). Also, extracting more classes yielded very small class sizes (smallest class was n = 58 in depression). We thus decided to keep the 2-class solution instead.

When we extracted the 3-class solutions in distress and resilience, although the AIC and BIC values were slightly better than of the 2-class solutions’, the VLMR LRT and LMR ALRT tests were not significant. Also, the sample sizes in the smallest classes were very small (n = 6 in distress, and n = 18 in resilience). Thus, we decided the use the 2-class solutions in all three measures.

We replicated the 2-class solutions with at least three sets of random starts as recommended in all three measures [2]. All model runs replicated the best log-likelihood values (Table S2, S3). The estimated loadings of wave 1 data on the latent slope factors in depressive symptoms was 4.184 (*SE* = 2.744, *p* > .1), and in distress was -4.302 (*SE* = 2.769, *p* > .1). Subsequently, we ran models using the 3-step method and predicted the latent classes with the measured covariates, but the models were too underpowered to estimate reliable coefficients. Therefore, further predictive analyses were done on the saved class memberships.

| **Table S1** Descriptive statistics of depressive symptoms, distress, and resilience scores used for latent trajectory estimation | | | | | |  |
| --- | --- | --- | --- | --- | --- | --- |
|  | *N* | Minimum | Maximum | Mean | *SD* | |
| Depressive symptoms 0 | 294 | 0.00 | 2.50 | 0.417 | 0.406 | |
| Depressive symptoms 1 | 287 | 0.00 | 2.50 | 0.546 | 0.463 | |
| Depressive symptoms 2 | 282 | 0.00 | 2.40 | 0.443 | 0.422 | |
| Distress 0 | 298 | 0.20 | 3.20 | 1.177 | 0.483 | |
| Distress 1 | 291 | 0.40 | 3.20 | 1.397 | 0.541 | |
| Distress 2 | 288 | 0.40 | 2.90 | 1.127 | 0.457 | |
| Resilience 0 | 289 | 1.17 | 5.00 | 3.420 | 0.672 | |
| Resilience 1 | 286 | 1.00 | 5.00 | 3.367 | 0.685 | |
| Resilience 2 | 286 | 1.00 | 5.00 | 3.399 | 0.674 | |
| *Note.* Depressive symptoms and distress scores were divided by 10 to keep the values between 0 to 10 to help model convergence. *SD* = standard deviation. | | | | | |  |

| **Table S2** Model parameters and results of the 2-class CPGMM and LCGM models | | | | | | | |
| --- | --- | --- | --- | --- | --- | --- | --- |
| Variable | Best Log-Likelihood | AIC | BIC | Entropy | VLM RLR | LMR ALRT | BLRT |
| Depressive symptoms^a^ | -205.796 | 447.592 | 515.421 | 0.757 | *p* < 0.001 | *p* < 0.001 | *p* < 0.001 |
| Distress^b^ | -553.509 | 1125.019 | 1158.962 | 0.689 | *p* = 0.011 | *p* = 0.013 | *p* < 0.001 |
| Resilience^b^ | -760.152 | 1536.304 | 1566.426 | 0.778 | *p* < 0.001 | *p* < 0.001 | *p* < 0.001 |
| *Note.* AIC=Akaike information criterion; BIC=Bayesian information criterion; VLM RLT=Vuong-Lo-Mendell-Rubin likelihood ratio test; LMR ALRT=Lo-Mendell-Rubin adjusted likelihood ratio test; BLRT=bootstrap likelihood ratio test.  ^a^ Covariance Pattern Growth Mixture Model (CPGMM).  ^b^ Latent Class Growth Model (LCGM). | | | | | | | |

| **Table S3** Class proportions and mean intercept and slope results in the 2-class models | | | | |
| --- | --- | --- | --- | --- |
|  | Symptoms | Proportion of participants (%) | Mean latent intercept factor (*S.E.*) | Mean latent slope factor (*S.E.*) |
| Depressive symptoms | Low | 38.75% | 0.131 (0.027)*** | 0.006 (0.008) |
|  | High | 61.13% | 0.584 (0.039)*** | 0.042 (0.029) |
| Distress | Low | 71.15% | 0.993 (0.041)*** | -0.053 (0.030)+ |
|  | High | 28.85% | 1.638 (0.097)*** | -0.048 (0.035) |
| Resilience | Low | 31.98% | 2.704 (0.086)*** | 0.024 (0.037) |
|  | High | 68.03% | 3.749 (0.045)*** | -0.032 (0.022) |
| *Note. S.E.* = standard error. + *p* < .1. *** *p* < .001. | | | | |

References

[1] Von Hippel PT. New confidence intervals and bias comparisons show that maximum likelihood can beat multiple imputation in small samples. Structural Equation Modeling: A Multidisciplinary Journal. 2016;23(3):422-37.

[2] Van De Schoot R, Sijbrandij M, Winter SD, Depaoli S, Vermunt JK. The GRoLTS-checklist: guidelines for reporting on latent trajectory studies. Structural Equation Modeling: A Multidisciplinary Journal. 2017;24(3):451-67.

[3] Formánek T, Csajbók Z, Wolfová K, Kučera M, Tom S, Aarsland D, et al. Trajectories of depressive symptoms and associated patterns of cognitive decline. Scientific reports. 2020;10(1):20888.

[4] Cermakova P, Csajbók Z. Household crowding in childhood and trajectories of depressive symptoms in mid-life and older age. Journal of Affective Disorders. 2023;340:456-61.

[5] Muthén B, Muthén L. Mplus. In: Handbook of item response theory, Chapman and Hall/CRC; 2017, p. 507-18.

[6] McNeish D, Harring J. Covariance pattern mixture models: Eliminating random effects to improve convergence and performance. Behavior Research Methods. 2020;52:947-79.

[7] Akaike H. Information theory and an extension of the maximum likelihood principle. In: Selected papers of hirotugu akaike, Springer; 1998, p. 199-213.

[8] Schwarz G. Estimating the dimension of a model. The annals of statistics. 1978:461-4.

[9] Vuong QH. Likelihood ratio tests for model selection and non-nested hypotheses. Econometrica: journal of the Econometric Society. 1989:307-33.

[10] Lo Y, Mendell NR, Rubin DB. Testing the number of components in a normal mixture. Biometrika. 2001;88(3):767-78.

[11] McLachlan GJ, Lee SX, Rathnayake SI. Finite mixture models. Annual review of statistics and its application. 2019;6:355-78.
